# Athleticism and sex impact neural processing of sound

**DOI:** 10.1038/s41598-022-19216-2

**Published:** 2022-09-07

**Authors:** Jennifer Krizman, Silvia Bonacina, Danielle Colegrove, Rembrandt Otto-Meyer, Trent Nicol, Nina Kraus

**Affiliations:** 1 Auditory Neuroscience Laboratory,; 2 Department of Communication Sciences and Disorders, Northwestern University, Evanston, IL 60208 USA; 3 Department of Neurobiology, Northwestern University, Evanston, IL 60208 USA; 4 Department of Otolaryngology, Northwestern University, Chicago, IL 60611 USA; 5 Department of Sports Medicine, Northwestern Medicine, Chicago, IL 60611 USA

**Keywords:** Neurophysiology, Sensory processing

## Abstract

Biology and experience both influence the auditory brain. Sex is one biological factor with pervasive effects on auditory processing. Females process sounds faster and more robustly than males. These differences are linked to hormone differences between the sexes. Athleticism is an experiential factor known to reduce ongoing neural noise, but whether it influences how sounds are processed by the brain is unknown. Furthermore, it is unknown whether sports participation influences auditory processing differently in males and females, given the well-documented sex differences in auditory processing seen in the general population. We hypothesized that athleticism enhances auditory processing and that these enhancements are greater in females. To test these hypotheses, we measured auditory processing in collegiate Division I male and female student-athletes and their non-athlete peers (total n = 1012) using the frequency-following response (FFR). The FFR is a neurophysiological response to sound that reflects the processing of discrete sound features. We measured across-trial consistency of the response in addition to fundamental frequency (F0) and harmonic encoding. We found that athletes had enhanced encoding of the harmonics, which was greatest in the female athletes, and that athletes had more consistent responses than non-athletes. In contrast, F0 encoding was reduced in athletes. The harmonic-encoding advantage in female athletes aligns with previous work linking harmonic encoding strength to female hormone levels and studies showing estrogen as mediating athlete sex differences in other sensory domains. Lastly, persistent deficits in auditory processing from previous concussive and repetitive subconcussive head trauma may underlie the reduced F0 encoding in athletes, as poor F0 encoding is a hallmark of concussion injury.

## Introduction

For the auditory brain, sex matters. The female brain responds to sounds faster and more robustly than males^1–3^. Animal models suggest that these sex differences in auditory processing are tied to hormone levels in females^4–7^, with the greatest differences occurring when female estrogen levels are highest^8^. Consistent with a tethering of hormone levels and auditory processing, in humans, differences between males and females are minimal during early childhood^9^ but begin to diverge during puberty, when differences in sex hormones are initiated ^10–12^, and continue to become more distinct into young adulthood^9,13^.

In addition to sex effects on auditory processing, the auditory brain is shaped by experience^14–18^. Auditory enrichment, such as speaking multiple languages or playing a musical instrument, enhances processing of sound features important for that experience^19–25^, while auditory impoverishment, such as linguistic deprivation associated with low socioeconomic status (SES), can lead to poorer processing of sound features and increased neural noise^26^, which is the random, intrinsic fluctuations in the brain that are not in response to an external stimulus. The neural noise differences are presumed to arise from differences in overall health, as low-SES individuals tend to be exposed to higher ambient environmental noise levels and eat food lower in nutritional value compared to high-SES individuals^27,28^. In contrast, reduced neural noise levels have been observed in collegiate athletes^29^, extraordinarily healthy individuals whose intense physical training and conditioning to maximize performance in their sport^30,31^ is hypothesized to underlie this reduction in neural noise.

While it is known that sports participation can reduce neural noise, it is unknown whether it can enhance auditory processing of specific sound features, similar to the way that auditory processing enhancements through lifelong bilingualism or music training manifest. We hypothesize that because athletes constantly hone their auditory system to communicate and react in noisy settings^32–35^, enhanced processing of discrete sound features should be seen in the adult athlete brain. Given that sex differences in auditory processing are pervasive post-puberty, it is necessary to determine whether sports participation affects male and female athletes differently. In athletes, sex differences are known to exist in visual processing^36,37^, skeletal injury rates and severity^38,39^, and risk, symptoms, and severity of a sports-related concussion^40,41^. Evidence suggests estrogen plays a role in these athlete sex differences^42–46^, similar to the sex differences seen in auditory processing. Because estrogen leads to more robust and faster auditory processing in females, we hypothesized that athlete-related enhancements in auditory processing are magnified in female athletes. That is, athletic performance accentuates the sex differences by further boosting auditory processing in female athletes.

To test the hypotheses that athleticism enhances auditory processing and that it leads to greater enhancements in female athletes compared to male athletes, we measured auditory processing in male and female Division I student-athletes and non-athletes using the frequency-following response (FFR), a neurophysiological response to sound that reflects the processing of sound details and is generated predominantly in the auditory midbrain^47–50^. The FFR captures microsecond-fast neural activity in response to distinct sound features in speech, such as the fundamental, or lowest, frequency as well as harmonics of the fundamental, which differentially combine to form speech formants, that is, the frequencies that distinguish words like ‘dad’ and ‘bad’^21,51,52^. FFRs were recorded in alternating polarity, in which the stimulus was presented in one polarity and a second polarity that was 180 degrees out of phase with the first polarity. Doing so allowed us to bias responses to specific frequency components. By adding the two polarities, we generate an envelope response (FFR_ENV_), which biases the fundamental, whereas, by subtracting the two polarities, we generate a temporal fine structure response (FFT_TFS_), which biases the higher frequencies^52,53^. We sought to determine whether sex and athletic experience affected these components differently. We compared the male and female athletes and non-athletes on frequency-encoding strength over the fundamental frequency, frequencies corresponding to the first formant (i.e., the lowest band of prominent frequencies contributing to the phonetic content of the sound), and frequencies between the first and second formants that are captured within the FFR. In non-athletes, encoding of these frequencies is known to be greater in females than males^1,9,13^. We predicted that athletic experience would enhance processing of these frequencies in both males and females (i.e., athlete main effect), but that the enhancement would be greater for female athletes than male athletes (i.e., sex by athlete interaction). We also compared participants on the across-trial replicability of their FFRs, a measure of how consistent the response is over time. Given that consistency of the response can be impacted by neural noise^26,52^, we predicted that athletes would have greater across-session response consistency than non-athletes, in line with previous findings that athletes have brains with less neural noise^29^. Previous studies have also found that response consistency does not differ between males and females^13^; we therefore predicted that it would not show a sex difference here.

To determine whether any observed differences were specific to these FFR components or reflected general differences between athletes and non-athletes in auditory processing, a follow-up analysis was run comparing these four groups on timing of 6 peaks, previously defined in this response^1,54^. The effect of sex on these absolute latencies has been well established, with females having earlier latencies than males^1,9,55,56^, consistent with earlier studies using simpler stimuli^2,57,58^ (although in response to complex sounds, the sex difference in peak latency emerges across development, with many timing differences seen in adulthood not present in early childhood^9^). We had no a-priori expectations for what would be observed between athletes and non-athletes.

## Results

In summary, across-trial response consistency and encoding of the first formant (F1) and high frequencies (HF) were higher in athletes compared to non-athletes, while encoding of the fundamental frequency (F0) was lower in athletes. The athlete harmonic enhancements were driven by higher levels of encoding in the female athletes. The sex differences and polarity effects were in line with previous findings, with females having more robust encoding of the F0, F1, and HF than males and the FFR_ENV_ responses being larger and more consistent than the FFR_TFS_ responses. Interestingly, with the addition of athletes, a new sex difference emerged: females had more consistent responses than males.

### Response consistency (RC)

**Figure 1:**
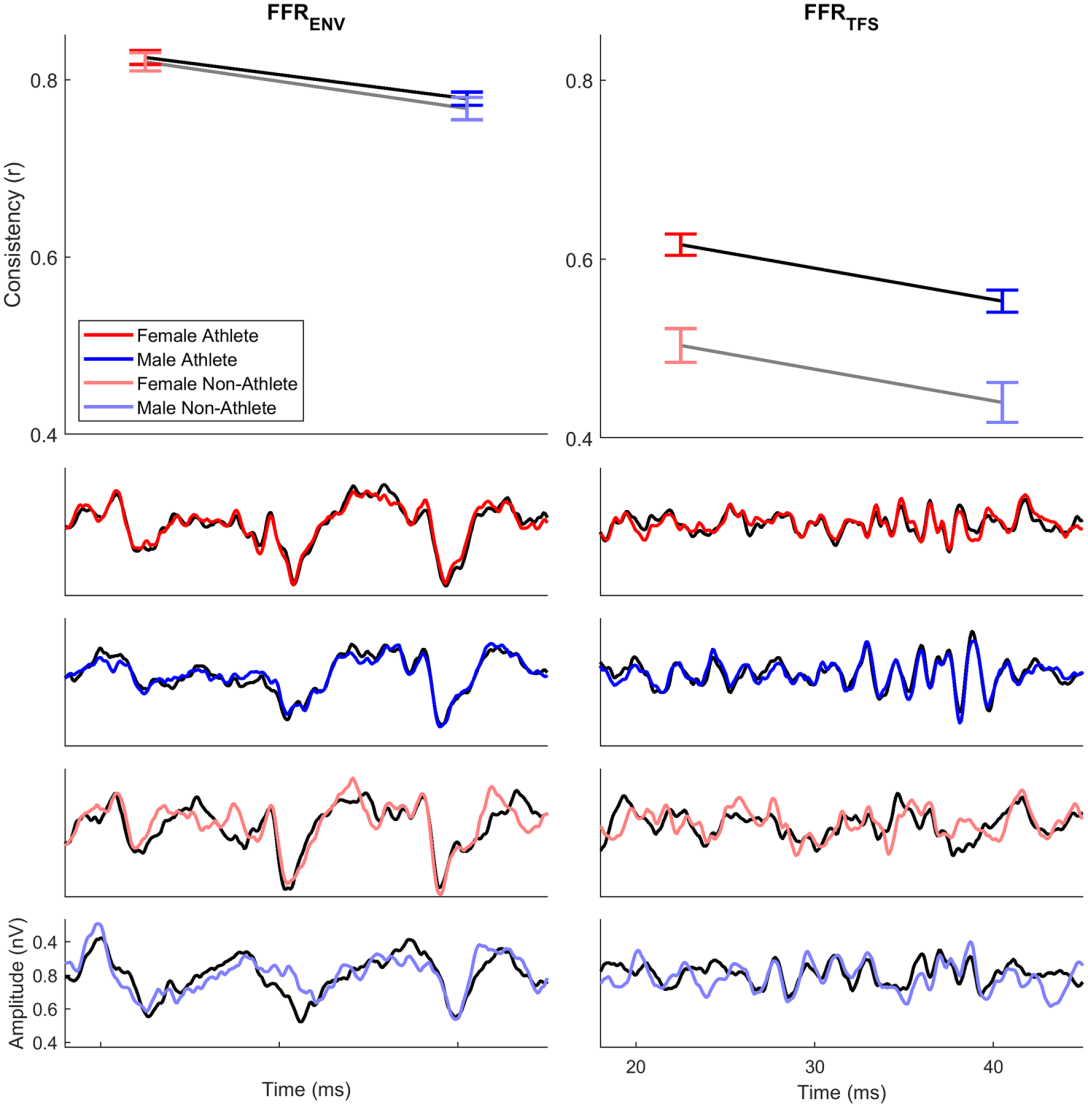
Response consistency (RC) differences in female and male athletes and non-athletes. Across-trial consistency of the FFR_ENV_ (left) and FFR_TFS_ (right) was greater for athletes (black lines of line plots) compared to non-athletes (gray lines of line plots). This RC difference was greater in the FFR_TFS_, with female athletes (red) and male athletes (blue) having greater consistency than female non-athletes (pink) and male non-athletes (light blue). From top to bottom, the waveforms in the bottom four plots display averages of the first half of the recording (black) and second half of the recording (red, blue, pink, and light blue) of a representative female athlete, male athlete, female non-athlete, and male non-athlete. Greater differences between the two waveforms indicates poorer RC, while greater similarity indicates higher RC.

**Figure 2:**
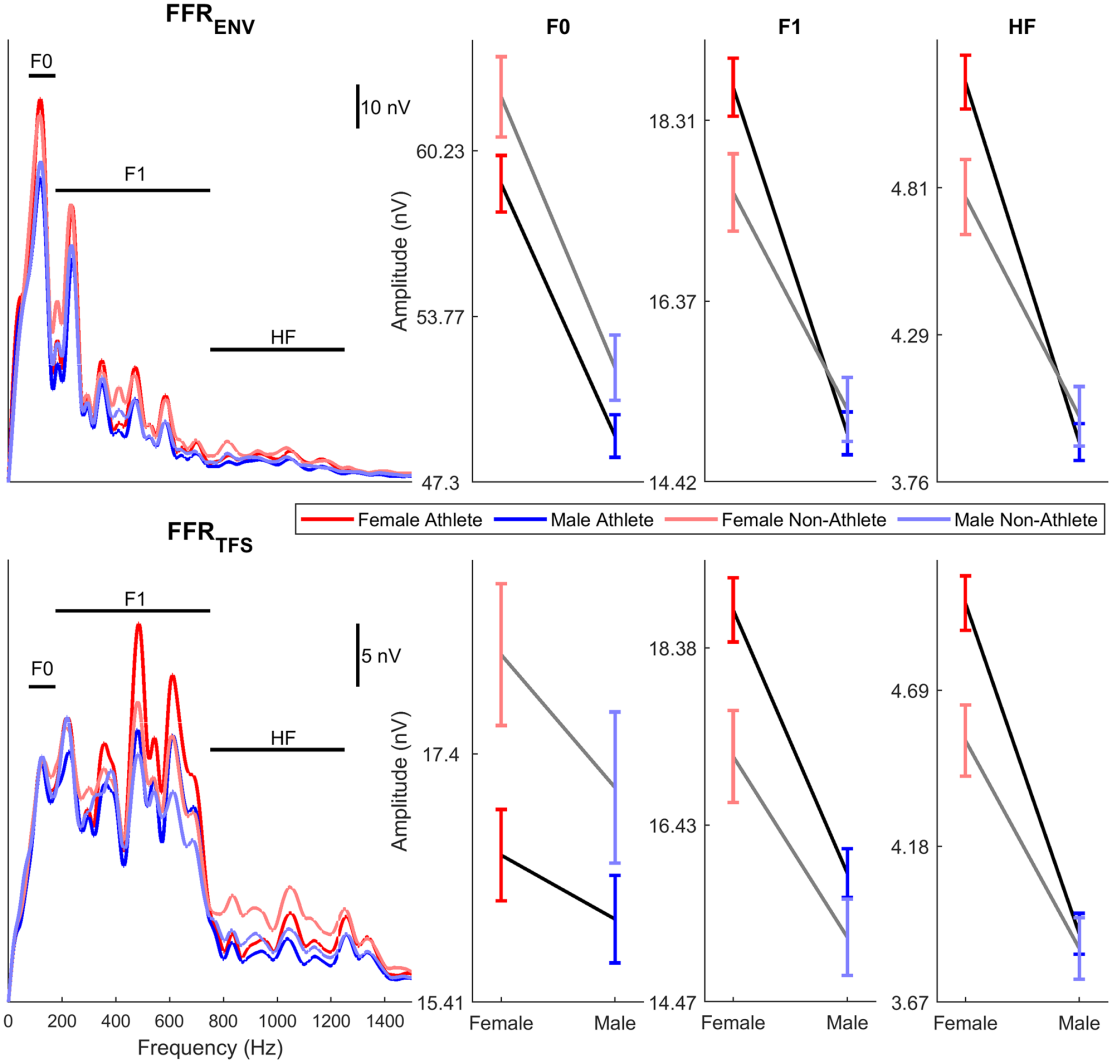
Frequency encoding differences in female and male athletes and non-athletes. Three frequency regions were evaluated, corresponding to the fundamental frequency (F0), frequencies within the first formant (F1), and higher frequencies that are above the first formant but within the phase-locking capabilities of the midbrain (HF). The frequencies corresponding to these regions are indicated by the black horizontal bars over the FFR_ENV_ (top left) and FFR_TFS_ (bottom left) spectra. For F0 encoding, athletes (black lines) had smaller responses than non-athletes (gray lines), for both polarities, regardless of sex (left line plots). Encoding of F1, was stronger in the athletes relative to non-athletes for both polarities. This difference was driven by greater F1 encoding in female athletes (red) than female non-athletes (pink), while male athletes (blue) were matched with non-male athletes (light blue) on F1 encoding (middle line plots). A similar effect was seen in HF encoding. (right line plots).

Across-trial consistency was higher for athletes compared to non-athletes (athletes r value: 0.692 ± 0.165; non-athletes r value: 0.637 ± 0.169, Fig. 1; see Table 1 for RMANOVA statistics). This difference in consistency was driven by greater differences between athletes and non-athletes for FFR_TFS_ (athletes: 0.583 ± 0.232; non-athletes: 0.477 ± 0.249; t(1008) = 6.431; p < 0.0005; d = 0.446) than for FFR_ENV_ (athletes: 0.801 ± 0.147; non-athletes: 0.798 ± 0.139; t(1008) = 0.497; p = 0.619; d = 0.034; Fig. 2), as indicated by the polarity by athlete status interaction.

**Table 1:** RMANOVA statistics. Degrees of freedom are (1, 1008). Significant differences are bolded and trending differences are italicized.

		F	*p*	η_p_^2^
**Response Consistency (RC)**	**Athlete**	**20.178**	** < .0005**	**.020**
	**Sex**	**32.316**	** < .0005**	**.031**
	**Polarity**	**1783.763**	** < .0005**	**.639**
	**Sex × Polarity**	**6.509**	**.011**	**.006**
	**Athlete × Polarity**	**31.738**	** < .0005**	**.031**
	Athlete × Sex	0.012	.914	0
	Athlete × Sex × Polarity	0.263	.608	0
**High Frequency Amplitude (HF)**	**Athlete**	**4.855**	**.028**	**.005**
	**Sex**	**107.308**	** < .0005**	**.096**
	**Polarity**	**11.024**	** < .0005**	**.011**
	*Sex × Polarity*	*2.823*	*.093*	*.003*
	Athlete × Polarity	0.882	.348	.001
	**Athlete × Sex**	**5.97**	**.015**	**.006**
	Athlete × Sex × Polarity	0.302	.583	0
**F1 Amplitude (F1)**	**Athlete**	**5.845**	**.016**	**.006**
	**Sex**	**68.560**	** < .0005**	**.064**
	Polarity	0.877	.349	.001
	Sex × Polarity	2.265	.133	.002
	*Athlete × Polarity*	*3.629*	*.057*	*.004*
	*Athlete × Sex*	*3.022*	*.082*	*.003*
	Athlete × Sex × Polarity	0.394	.530	0
**F0 Amplitude (F0)**	**Athlete**	**9.223**	**.002**	**.009**
	**Sex**	**58.720**	** < .0005**	**.055**
	**Polarity**	**3646.312**	** < .0005**	**.783**
	**Sex × Polarity**	**54.421**	** < .0005**	**.051**
	Athlete × Polarity	1.768	.184	.002
	Athlete × Sex	0.198	.656	0
	Athlete × Sex × Polarity	0.005	.946	0

Additionally, there were main effects of polarity and sex, as well as a polarity by sex interaction. FFR_ENV_ responses had greater consistency than FFR_TFS_ responses (FFR_ENV_: 0.800 ± 0.145; FFR_TFS_: 0.552 ± 0.242) and females had higher response consistency than males (females: 0.701 ± 0.161; males: 0.650 ± 0.171). This sex difference was greater for FFR_ENV_ than for FFR_TFS_ (females, FFR_ENV_: 0.823 ± 0.140; males, FFR_ENV_: 0.776 ± 0.145; t(1008) = 6.505; p < 0.0005; d = 0.409; females, FFR_TFS_: 0.579 ± 0.233; males, FFR_TFS_: 0.525 ± 0.248; t(1008) = 3.567; p < 0.0005; d = 0.224). RC means and standard deviations broken down by the four groups (female athlete, male athlete, female non-athlete, male non-athlete) at each polarity are reported in Table 2.

**Table 2:** Means and standard deviations for the four groups on the four measures in the two polarities.

		Athlete		Non-Athlete	
		Female	Male	Female	Male
FFR_ENV_	F0 Amplitude (nV)	58.941 ± 20.367	49.100 ± 16.190	62.331 ± 20.370	51.764 ± 14.295
	F1 Amplitude (nV)	18.666 ± 5.758	14.947 ± 4.457	17.535 ± 5.395	15.205 ± 3.853
	High Frequency Amplitude (nV)	5.190 ± 1.770	3.904 ± 1.283	4.779 ± 1.740	3.996 ± 1.197
	Response Consistency (r)	0.825 ± 0.144	0.779 ± 0.146	0.820 ± 0.133	0.768 ± 0.142
FFR_TFS_	F0 Amplitude	16.592 ± 6.749	16.079 ± 6.795	18.197 ± 7.376	17.133 ± 6.792
	F1 Amplitude	18.801 ± 6.535	15.894 ± 5.237	17.183 ± 6.607	15.187 ± 4.72
	High Frequency Amplitude	4.974 ± 1.635	3.897 ± 1.297	4.526 ± 1.509	3.849 ± 1.120
	Response Consistency	0.616 ± 0.217	0.553 ± 0.241	0.504 ± 0.245	0.440 ± 0.251

### High frequency (HF) encoding

HF encoding differences between athletes and non-athletes were similar to the effects seen in RC. Athletes had greater HF encoding than non-athletes (athletes: 4.460 ± 1.457 nV; non-athletes: 4.341 ± 1.348 nV; Fig. 2; see Table 1 for all RMANOVA statistics). There was a sex by athlete interaction, showing that female athletes had higher encoding than female non-athletes (female athletes: 5.082 ± 1.512 nV; female non-athletes: 4.653 ± 1.473 nV; t(507) = 3.039, p = 0.002, d = 0.286), while males did not differ (male athlete: 3.900 ± 1.148 nV; male non-athlete: 3.922 ± 1.024 nV; t(501) = 0.193; p = 0.847; d = 0.020).

There were also main effects of sex and polarity, with females having higher HF responses than males (females: 4.939 ± 1.512 nV; males: 3.906 ± 1.117 nV), and FFR_ENV_ being larger than FFR_TFS_ (FFR_ENV_: 4.494 ± 1.637 nV; FFR_TFS_: 4.358 ± 1.516 nV). HF means and standard deviations for the four groups at each polarity are reported in Table 2.

### First formant (F1) encoding

F1 encoding was greater in athletes compared to non-athletes (athletes: 16.991 ± 5.059 nV; non-athletes: 16.435 ± 4.753 nV; Fig. 2; see Table 1 for all RMANOVA statistics). Similar to the sex by athlete effects seen for HF encoding, there was also a trending interaction between sex and athlete status for F1 encoding, suggesting the athlete enhancement was driven by female athletes having more robust responses than non-athlete females (female athletes: 18.733 ± 5.347 nV; female non-athletes: 17.359 ± 5.29; t(507) = 2.740; *p* = 0.006; d = 0.258) while male athletes and non-athletes did not differ (male athletes: 15.421 ± 4.211; male non-athletes: 15.196 ± 3.581; t(501) = 0.537; *p* = 0.591; d = 0.055).

Females had larger F1 responses than males (females: 18.277 ± 5.362 nV; 15.364 ± 4.06 nV). F1 amplitude, however, did not differ between polarities (FFR_ENV_: 16.661 ± 5.293 nV; FFR_TFS_: 16.998 ± 6.041 nV). F1 means and standard deviations for the four groups at each polarity are reported in Table 2.

### Fundamental frequency (F0) encoding

In contrast to the athlete enhancements found for RC, HF encoding, and F1 encoding, F0 encoding was smaller in athletes than non-athletes (athletes: 35.045 ± 10.598 nV; non-athletes: 37.780 ± 10.668 nV; Fig. 2; see Table 1 for all RMANOVA statistics).

There was also a main effect of sex, with females having larger responses than males (females: 38.596 ± 11.451 nV; males: 33.056 ± 9.044 nV), as well as a main effect of polarity, where responses were larger for FFR_ENV_ (FFR_ENV_: 54.948 ± 18.955 nV; FFR_TFS_: 16.736 ± 6.909 nV). In addition to these main effects, there was an interaction of polarity by sex, which showed that the sex difference was greater for FFR_ENV_ (females: 60.066 ± 20.411 nV; males: 49.768 ± 15.766 nV; t(1010) = 8.976, *p* < 0.0005, d = 0.564) than for FFR_TFS_ (females: 17.125 ± 6.997 nV; males: 16.343 ± 6.803; t(1010) = 1.801, *p* = 0.072, d = 0.113). F0 means and standard deviations for the four groups at each polarity are reported in Table 2.

### Peak latencies

There was a main effect of sex on peak timing (F(1, 1006) = 125.898, *p* < 0.001, η_p_^2^ = 0.111), with females having earlier peaks than males (females: 25.822 ± 0.294 ms; males: 26.093 ± 0.345 ms; t(1010) = 13.455, *p* < 0.001, d = 0.846). Athleticism did not influence peak timing (F(1, 1006) = 0.787, *p* = 0.375, η_p_^2^ = 0.001). While there was an interaction between sex and athletes (F(1, 1006) = 4.599, *p* = 0.032, η_p_^2^ = 0.005), only the expected sex differences were evident. Female athletes (25.801 ± 0.283 ms) were earlier than male athletes (26.099 ± 0.360 ms; t(715) = 12.234, *p* < 0.001, d = 0.915) and male non-athletes (26.078 ± 0.299 ms; t(464) = 9.243, *p* < 0.001, d = 0.964). Female non-athletes (25.865 ± 0.312 ms) were earlier than male athletes (t(544) = 7.292, *p* < 0.001, d = 0.675) and male non-athletes (t(293) = 5.885, *p* < 0.001, d = 0.693). Female athletes and non-athletes (t(507) = 2.339, *p* = 0.01, d = 0.220) and male athletes and non-athletes (t(501) = 0.599, *p* = 0.275, d = 0.062) did not differ. There were no peak by sex (F(5, 5030) = 1.403, *p* = 0.220, η_p_^2^ = 0.001), peak by athlete (F(5, 5030) = 0.928, *p* = 0.461, η_p_^2^ = 0.001) or peak by sex by athlete (F(5, 5030) = 0.176, *p* = 972, η_p_^2^ = 0) interactions.

## Discussion

We compared collegiate male and female student-athletes participating in a Division I (i.e., the highest level of collegiate athletics) sport to their non-athlete peers on auditory processing using the FFR. We found that athletes showed increased across-trial consistency of the response. Athletes exhibited enhanced encoding of harmonic frequencies (F1 and HF), and these effects were especially pronounced in female athletes. F0 encoding, however, was smaller in athletes than non-athletes, contrary to our predictions. We replicated well-established polarity^52,53^ and sex differences^1,13,52^ but also found that response consistency is higher in females than males, contrary to previous results^13^. Interestingly, we found that while females had earlier peak latencies than males, consistent with previous findings^1,9,56,57^, athlete status had no effect on FFR peak timing, suggesting that the enhancements are specific to harmonic encoding and response consistency, along with the previous reports of lower noise levels^29^.

### Athletes have more consistent responses to sound than non-athletes

Athletes have quieter brains than non-athletes, driven by lower noise levels in the athlete brain^29^. Neural noise can interfere with auditory processing, leading to smaller and less consistent responses^26,52,59^. That is, noise can obscure the encoding of the distinct features of a response, and given the stochastic nature of noise, what is obscured one time the sound is heard could be different from what is obscured the next time. The lower levels of neural noise in athletes yield more consistent responses, given that the lower noise levels lead to less interference in the brain’s response to sound from one trial to the next. Having a quieter brain and a more consistent response is advantageous for athletic competition because it enables the listener to respond to important auditory cues in their competition.

While we predicted the observed difference between athletes and non-athletes in the consistency of their responses, the sex difference ran counter to our expectations, as we have not seen this component differ between males and females previously^13^. Because about two-thirds of our females were athletes, the higher number of female athletes might have brought out this sex difference in response consistency. Alternatively, the large number of participants included in these analyses may have allowed this effect to emerge. Thus, while we can conclude that athletes have more consistent neural responses to sound than non-athletes, the observed sex differences in this measure warrants further investigation.

### Athleticism enhances harmonic encoding, especially in female athletes

Overall, we found that athletes have stronger encoding of a sound’s harmonics. Auditory cues are known to be important for both team and individual sports^60^. These cues provide information about the direction or speed of a moving object, such as a soccer ball or volleyball^35^, guide motor action of the athlete^32^, give feedback about body positioning^61^, or signal to the athlete that they can initiate play (e.g., a whistle). Speech, in particular, plays a crucial role in many sports. During a football game, the rest of the offense must listen for the commands shouted by the quarterback while ignoring the screams and cheers of the crowd. Volleyball players must communicate among one another to know, for example, who will pass a served ball. A basketball team listens for their point guard to tell them which plays to run. And these athletes, as well as those in individual sports (e.g., singles tennis, swimming), must listen for their coach’s feedback being shouted from the sideline. Thus, athletes must hone their auditory systems to pick up on these important cues and signals. We speculate athletes do this by enhancing their encoding of the harmonic frequencies, which could aid in identifying the important incoming auditory information, especially speech. The harmonic frequencies are what comprise speech formants, which play a major role in distinguishing speech sounds, and ultimately words, from one another^62,63^. Better encoding of these frequencies, then, could aid communication during competition.

Although there was a main effect of athlete status on harmonic encoding, there was also an interaction between sex and athlete status, with post-hoc tests showing the enhancement was greatest for female athletes. Notably, harmonic encoding is the aspect of auditory processing most strongly tethered to hormone levels, with the greatest harmonic encoding occurring in female rodents when estrogen levels were highest^8^. Consistent with other studies finding that estrogen can lead to sex-specific enhancements in sensory processing^36,37,46^, estrogen may mediate the pronounced harmonic encoding enhancements seen in female athletes. Additionally, a majority of our male athletes participate in football, a sport that has a high rate of head trauma. It is possible that the higher percentage of males in a contact sport are leading to a dampening of the harmonic enhancement in males. Future studies should examine the role that contact level plays on the athlete harmonic enhancement.

### Athletes have smaller F0 encoding: an effect of subconcussion?

Interestingly, while we found that harmonic (F1 and HF) encoding was greater in athletes than non-athletes, we found the opposite effect for encoding of the fundamental frequency. The F0 reduction seen in the athletes could stem from two potential sources, which are not mutually exclusive: (1) athletes with a history of concussion have a lasting reduction in F0 encoding; (2) an accumulation of subconcussive head impacts in the athlete population have fostered an overall decline of F0 encoding in this group. F0 encoding impairments are a hallmark of concussion in acutely injured individuals^64,65^. Despite some recovery of the F0 as the individual recovers^64^, a lingering deficit can remain^66^. Athletes, especially, those participating in contact or collision sports, are at greater risk than non-athletes of sustaining a concussion^67^. But, even more common for these athletes, is that they regularly sustain subconcussive impacts^68,69^. These impacts can be to the head or body and are below the threshold that causes overt concussion symptoms. Although subconcussive impacts may be insufficient to cause damage following a single event, neurodegeneration from repetitive hits can accrue over time^70–73^. The lingering effects of concussion combined with the gradual accumulation of subconcussive hits could, therefore, lead to poorer F0 encoding in a presumably healthy population. Given that about three-quarters of our athletes participated in collision (football), contact (soccer, field hockey, lacrosse, wrestling), and limited-contact (baseball, softball, basketball, volleyball, diving) sports, these athletes may have sustained more head impacts (both concussive and subconcussive) than their non-athlete peers^68,74^. Previous studies have found deficits in auditory processing following head injury^75–78^, but the effects of head trauma on auditory processing are often overlooked^79^. Future work should explore links between auditory-processing deficits and head trauma further.

### Conclusions

In conclusion, athletic experience impacts the neural processing of sound. First, athletes have more consistent responses to sound. How consistently a brain responds to sound each time it is heard is partly influenced by the levels of noise in the brain^26,52^. The lower noise levels^29^ enable the athlete brain to respond more consistently to a sound over time. Second, athletes have more robust encoding of the harmonic frequencies of sound, providing them important information about the identity of the sound or the meaning of words. This harmonic encoding enhancement was more prominent for the female athletes, suggesting a role of estrogen in mediating this enhancement. Lastly, encoding of the fundamental frequency was poorer in athletes compared to non-athletes, which may indicate a persistent effect of head trauma in these individuals. Future research should investigate the link between decreased F0 encoding and long-term effects of head impacts in healthy athletes.

## Methods

### Participants

Participants were 1012 (509 female) college-aged individuals recruited from a Midwestern U.S. university (M ± SD age = 19.432 ± 1.186 years, age range = 17.5–22 years). Of these, 717 (340 female) were National Collegiate Athletic Association (NCAA) Division I student-athletes and 295 (169 female) were non-athletes (see Table 3 for breakdown by sport). Division I sports are the highest level of intercollegiate athletics sanctioned by the NCAA. Comparisons were made among female athletes (19.322 ± 1.097 years), male athletes (19.515 ± 1.105 years), female non-athletes (19.539 ± 1.383 years), and male non-athletes (19.339 ± 1.339 years). The four groups were matched on age (F(3, 1011) = 2.324, *p* = 0.073; η_p_^2^ = 0.007). All procedures were approved by the University’s Institutional Review Board (IRB) and completed according to the rules and regulations set forth by this committee and the Declaration of Helsinki. Participants provided informed consent to participate and were compensated monetarily for their time.

**Table 3:** Breakdown of participants by sport and sex.

	Female	Male
Baseball	0	46
Basketball	18	19
Cross Country	31	0
Fencing	37	0
Field Hockey	44	0
Football	0	182
Golf	14	12
Lacrosse	45	0
Soccer	46	38
Softball	27	0
Swim & Dive	45	35
Tennis	25	15
Volleyball	8	0
Wrestling	0	30
Non-Athlete	169	126

### Stimulus and recording parameters

The frequency-following response (FFR) was elicited by a synthesized speech sound^80^, ‘da’, described previously in detail^1,8^. Briefly, the ‘da’ is 40-ms in duration, beginning with a noise burst, followed by a formant transition between the ‘d’ and the ‘a’. The fundamental frequency (F0) and first three formants (F1, F2, F3) change linearly over the formant transition from 103–125 Hz, 220–720 Hz, 1700–1240 Hz, and 2580–2500 Hz, respectively. The fourth (F4, 3600 Hz) and fifth (F5, 4500 Hz) are constant. The ‘da’ was presented monaurally through a shielded insert earphone (ER-3A, Natus Medical Inc., Mundelein, IL) to the right ear via alternating polarity at a rate of 10.9 Hz and intensity of 80 dB SPL. To collect the FFRs, the participant sat comfortably in a darkened, quiet room while the FFR was passively recorded by Ag/AgCl electrodes affixed to the participant in an ipsilateral vertical montage, with active at Cz, reference on the right ear lobe, and ground on the forehead. FFRs were collected in Bio-logic Navigator Pro AEP (Natus Medical Inc., Mundelein, IL) using an epoch window that began 15.8 ms prior to stimulus onset to capture background neural activity. Online, responses were filtered from 100 to 2000 Hz and artifact rejected at ± 23,800 nV. For each participant, two averaged waveforms were recorded successively, each containing 3000 artifact-free responses, 1500 of each polarity. For the frequency measures described below, these two FFRs were combined to create an averaged response of 6000 artifact-free sweeps.

### Data processing

FFRs were generated in two ways: one in which responses from the two polarities are added together to accentuate the envelope (ENV) and lower-frequency components of the response, and one in which the responses are subtracted to accentuate higher-frequency temporal fine structure (TFS) components^52,53^. For each participant’s FFR_ENV_ and FFR_TFS_, we computed four measures over the 19.5–44.2 ms time region of the response, a region of the response that tracks the periodicity of the frequencies in the sound. Three of these were measures of averaged spectral amplitudes calculated over ranges encompassing the F0 (75–175 Hz), F1 (175–750 Hz), and frequencies between the first and second formant (750–1250 Hz, subsequently referred to as high frequencies, or HF) that are still within the phase-locking capabilities of the midbrain^81^, the primary generator of the FFR^47,50,82^. The fourth measure was the across-trial response consistency of the FFR, calculated by correlating the averages from the first half and last half of the recording session using a Pearson product-moment correlation, with r-values closer to 1 representing more consistent responses. Because the FFR_ENV_ response is low-frequency biased, the amplitude of the F0 is larger in this response than in the FFR_TFS_ response, while differences in F1 and HF amplitude between the two tend to be smaller^52,53^. Additionally, the low-frequency bias of the FFR_ENV_ response results in higher response consistency values compared to the FFR_TFS_^13^. Lastly, sex differences are greater in the FFR_ENV_ component compared to the FFR_TFS_ component^13^. We expected that the current analyses would yield results in line with these well-established polarity effects.

Peak latencies were determined for peaks V, A, D, E, F, and O, occurring at approximately 6.6 ms, 7.6 ms, 22.5, 31, 39.5, and 48 ms, respectively. Peaks were picked by an experienced peak picker based on previously established methods^1^. Peaks were checked by two expert pickers blind to participant group.

### Statistical analyses

For each FFR measure, except latency, we ran a 2 (polarity: FFR_ENV_ v. FFR_TFS_) × 2 (sex: male v. female) × 2 (group: athlete v. non-athlete) repeated-measures analysis of variance (RMANOVA) in SPSS (Version 28, IBM Inc., Chicago, IL). Latencies were compared between groups on the FFR_ENV_ response using a 6 (peak: V, A, D, E, F, O) × 2 (sex: male v. female) × 2 (group: athlete v. non-athlete) repeated-measures analysis of variance (RMANOVA) in SPSS. While across group analyses considered each peak separately, means and standard deviations in the text were reported as an average of the 6 peaks for each participant since there were no interactions with peak. Bonferroni-corrected post-hoc t-tests, using a *p* < 0.0083 criterion, were run on any significant interaction to explore these effects further. While figures and descriptive statistics provide *r* values, these values were Fisher-transformed prior to analyzing to increase the normality of the distribution.

## Data Availability

Data and materials are available upon request by contacting the corresponding author (nkraus@northwestern.edu).
